# The definition of disabling fatigue in children and adolescents

**DOI:** 10.1186/1471-2296-6-33

**Published:** 2005-08-09

**Authors:** Tom Fowler, Pamela Duthie, Anita Thapar, Anne Farmer

**Affiliations:** 1 Department of Psychological Medicine, Wales College of Medicine, Cardiff University, UK; 2 Brynffynnon Child & Family Service Unit, Pontypridd, UK; 3 MRC Social, Genetic, Developmental Psychiatric Research Centre, Institute of Psychiatry, London, UK

## Abstract

**Background:**

Disabling fatigue is the main illness related reason for prolonged absence from school. Although there are accepted criteria for diagnosing chronic fatigue in adults, it remains uncertain as to how best to define disabling fatigue and Chronic Fatigue Syndrome (CFS) in children and adolescents. In this population-based study, the aim was to identify children who had experienced an episode of disabling fatigue and examine the clinical and demographic differences between those individuals who fulfilled a narrow definition of disabling fatigue and those who fulfilled broader definitions of disabling fatigue.

**Methods:**

Participants (aged 8–17 years) were identified from a population-based twin register. Parent report was used to identify children who had ever experienced a period of disabling fatigue. Standardised telephone interviews were then conducted with the parents of these affected children. Data on clinical and demographic characteristics, including age of onset, gender, days per week affected, hours per day spent resting, absence from school, comorbidity with depression and a global measure of impairment due to the fatigue, were examined. A narrow definition was defined as a minimum of 6 months disabling fatigue plus at least 4 associated symptoms, which is comparable to the operational criteria for CFS in adults. Broader definitions included those with at least 3 months of disabling fatigue and 4 or more of the associated symptoms and those with simply a minimum of 3 months of disabling fatigue. Groups were mutually exclusive.

**Results:**

Questionnaires were returned by 1468 families (65% response rate) and telephone interviews were completed on 99 of the 129 participants (77%) who had experienced fatigue. There were no significant differences in demographic and clinical characteristics or levels of impairment between those who fulfilled the narrower definition and those who fulfilled the broader definitions. The only exception was the reported number of days per week that the child was affected by the fatigue. All groups demonstrated evidence of substantial impairment associated with the fatigue.

**Conclusion:**

Children and adolescents who do not fulfil the current narrow definition of CFS but do suffer from disabling fatigue show comparable and substantial impairment. In primary care settings, a broader definition of disabling fatigue would improve the identification of impaired children and adolescents who require support.

## Background

Disabling fatigue in children and adolescents is an important clinical problem given that it is associated with severe functional impairment that has an impact upon the child's education and social development [1,2]. It is the main illness related reason for prolonged absence from school in young people [3]. It is also a presenting problem for 2% and a background problem in 11% of school children attending general paediatric clinics [4]. There is uncertainty about how to appropriately define disabling fatigue in children and adolescents. Reviews of disabling fatigue in children and adolescents highlight the lack of research in this age group in comparison to adults [2].

There have been several attempts to define disabling fatigue as a disorder in adults and perhaps the most accepted is the Center for Disease Control (CDC) definition [5]. This requires 6 months of disabling fatigue and 4 or more of the following symptoms; 1) impaired memory or concentration, 2) sore throat, 3) tender cervical axillary lymph nodes, 4) muscle pain, 5) multi-joint pain, 6) new headaches, 7) unrefreshing sleep, 8) post-exertion malaise. This has, to date, been the most widely used definition when reporting the prevalence of CFS and CFS like illness in children and adolescents [6-9].

A number of authors have raised the concern that the current criteria for CFS were designed for use in adults and that there has been relatively little work in assessing how appropriate these criteria are for children and adolescents [10-14]. Although a consensus document on CFS by the Royal Colleges [2] suggested that the criteria were applicable to children and adolescents it did also suggest the period of disabling fatigue required for a diagnosis of CFS should be reduced from 6 to 3 months.

The importance that these associated symptoms play in CFS and whether they contribute to defining a homogenous group of individuals with disabling fatigue has been increasingly questioned [15]. As the associated symptoms were originally identified by expert consensus there are growing calls for studies examining individuals with chronic unexplained fatigue to empirically derive a definition of CFS [16].

Given the current discussion about the role of the associated symptoms in CFS in adults, the lack of empirical testing about the appropriateness of the adult CFS criteria in children it seems pertinent to examine whether the clinical and demographic features of children and adolescents differ in those who fulfil CFS criteria and those who fulfil broader criteria for disabling fatigue. Evidence that those individuals with "adult like CFS" did differ significantly would give credence to the idea that adult CFS criteria do identify a specific group of fatigued individuals.

There is also evidence to suggest that the majority (two thirds) of adults who attend GP clinics regarding disabling fatigue lasting more then 6 months do not fulfil the CDC definition of CFS, but do have substantial associated impairment [17]. Given this it is also important to examine and compare the levels of impairment in children and adolescents who fulfil CFS criteria and those who fulfil broader definitions of disabling fatigue. The latter may represent an important group of individuals who require treatment and primary care support.

In this paper, we set out to identify children and adolescents with a lifetime ever episode of disabling fatigue in a population-based sample and test whether those who fulfilled a narrow definition were more severely impaired and different in terms of clinical and demographic characteristics than those who only fulfilled broader definitions of chronic disabling fatigue. Specifically, three groups of individuals with disabling fatigue were defined; those who had a similar symptom profile, duration and impairment as the CDC operational criteria for CFS in adults ("adult like CFS"), those who also had a similar symptom profile and impairment but whose fatigue, at time of interview, had lasted for a period of between 3–6 months disabling fatigue ("child like CFS") and those that did not fulfil the symptom profile for operationally defined CFS but who had had more than 3 months disabling fatigue ("3 months plus disabling fatigue").

## Methods

### Participants

Participants were identified from the population based twin register CaStANET (Cardiff Study of All Wales and North West England Twins). Previous studies have shown that the register is representative of the local population [18]. Initially 2259 twin pairs aged between 8–17 years old were identified from the register and a questionnaire package was sent to these twins and their parents. Families in which the parents reported that a child had experienced a period of disabling fatigue which had lasted for more than a few days and had caused interference with activities such as being able to go to school, or see friends or family, were selected for a standardised parental telephone interview. Ethical approval was granted by the Multi-Centre Research Ethics Committee (MREC) for Wales.

### Measures

The parental telephone interview is described in detail elsewhere [10]. The interview consisted of two parts. The first part obtained further details about the period(s) of fatigue.

To assess whether the young person's fatigue could be classified as "adult like CFS", "child like CFS" or "3 months plus disabling fatigue" enquiry was made about the associated symptoms that sometimes occur with fatigue [5]. These were poor memory and concentration, difficulty thinking, sore throats, tender lymph nodes, muscle pain, multiple joint pains, headaches, unrefreshing sleep, fatigubility, or post-exertion malaise associated with the period of fatigue. Items were rated as 'less than usual', 'same as usual', 'more than usual' or 'a lot more than usual' for the episode of fatigue. Those children whose parents reported "more than usual" or "a lot more than usual" for 4 or more of the associated symptoms required by the CDC definition of CFS in adults were classed as fulfilling the associated symptom requirement.

Questions were also asked about the duration of the fatigue, whether it was ongoing at the time of interview and whether it had been continuous or episodic (where the periods of fatigue were described as episodic the parent was asked to focus on the longest most debilitating period of fatigue for the remainder of the questions). The duration of the fatigue at the time of interview was used to classify the twins into the different categories regardless of whether the fatigue was currently ongoing. The nature and degree of impairment associated with the fatigue was assessed by enquiring about whether the twin needed to rest for at least 1 hour daily, and whether there was interference with school attendance, and/or usual leisure activities and with family and peer relationships. To be classed as suffering from "disabling fatigue" the twin was required to need to rest for at least 1 hr daily and for there to be a report of some interference in at least one of these areas.

Individuals who did not fulfil the associated symptom requirement but had experienced a period of disabling fatigue of greater than 3 months at the time of interview were classed as suffering from "three months plus disabling fatigue". Those who did fulfil the associated symptom requirement but had only experienced a period of between 3–6 months of the disabling fatigue at the time of interview were classed as suffering from "child like CFS" and those who has experienced a period of more than 6 months disabling fatigue were classed as suffering from "adult like CFS"

Information was also sought about the age of onset of the fatigue, the number of days per week affected by the fatigue, the number of hours per day that the affected child required to rest/sleep, whether the child or adolescent was absent from school due to the fatigue, and whether they were taken to visit a General Practioner with regard to the fatigue. A global measure of impairment was created by asking whether the fatigue had interfered with the following 4 areas; i) the child's schoolwork, ii) peer relations, iii) family relations and iv) their usual leisure pursuits. The possible response's to these questions were on a discrete adjective scale consisting of "Not much", "A little" or "A lot" and were scored as 0, 1, or 2 respectively. The answers to these questions were then summed to give the overall score which ranged from 0 to 8.

Parents were asked whether any diagnoses or explanation for the disabling fatigue had been offered by GP's or hospital specialists, when these had been visited. Their responses were recorded verbatim. This information was then reviewed by one of the authors (PD) who was blind to other information from the data collection and who determined those cases where the diagnosis could entirely explain the chronic fatigue in the twin. These cases were then excluded from further analysis.

The second part of the interview consisted of the depression section of the parent version Child and Adolescent Psychiatric Assessment (CAPA) [19]. This is a standardised, reliable psychiatric interview that assesses psychopathology in children and adolescents. The responses to this were used to generate DSM-IV defined diagnoses of major depression during the episode of fatigue.

### Analysis

As it was determined that the most important analysis to undertake was to assess whether those individuals who fulfilled just the broad definitions of disabling fatigue ("child like CFS" and "3 months plus disabling fatigue") differed from those who fulfilled the narrow definition ("adult like CFS") and to reduce multiple testing no comparisons were made between individual classed as suffering from "child like CFS" and those who had "3 months plus disabling fatigue". Two sets of comparisons were therefore made, the first between those individuals who had "adult like CFS" and those who had "child like CFS", the second between those individuals who had "adult like CFS" and "3 months plus disabling fatigue". Independent t-tests and chi-square tests were used as appropriate.

However, although the twin register is population based, each twin pair could potentially contributes two observations, and where this is the case the individual twin cannot be classed as statistically independent. Reduced variance due to the correlation between twins' scores can cause high false positive rates [20]. To adjust for this bias the data was treated as equivalent to a 2-stage cluster design with the twin pairs as the primary sampling unit [21]. Consequently, the survey analysis procedures of the statistical analysis package STATA Release 6 [22] were used to adjust the variances of all analyses to be equivalent to independently sampled pairs. Each family unit was classed as a clustering unit, with some clusters containing information from both twins, while others contained information from just one.

## Results

### Participants

Parents from 1468 families returned questionnaires (65% response rate). There were no significant socio-demographic differences between the responding and non-responding families [18]. The screening questionnaire identified one hundred and twenty nine children and adolescents who had experienced more than a few days of disabling fatigue and telephone interviews were undertaken on 99 (77%). For the remaining individuals parents had either not given permission to be contacted for a telephone interview when returning the questionnaires or when contacted did not wish to take part. Of the families who met the selection critieria there were 11 twin pairs where interviews were conducted about both twins. Following the interview, 3 participants whose parents reported diagnoses of Cerebral Palsy, Nephrotic Syndrome and Thalassaemia, were excluded from the analysis as it was felt that these disorders could entirely explain the presence of the disabling fatigue.

### Analysis

Twins classed as suffering from "3 months plus disabling fatigue" had an average duration of fatigue of 25.8 months (standard error of mean, SEM, 14.0) of which 27% still had ongoing fatigue at the time of interview. Twins classed as suffering from "adult like CFS" had an average duration of 23.5 months of fatigue (SEM 6.1) with 41% with ongoing fatigue at the time of interview. Those classed as suffering "child like CFS" had by definition between 3–6 months disabling fatigue, of which 27% had ongoing fatigue at the time of interview. Table 1 shows the male: female ratio, age of onset, days per week impaired during the worst period of fatigue, hours per day impaired by the fatigue, number of days absent from school per term during the worst period of fatigue and the global impairment score. It also presents the percentage in each group who visited a GP with regard to the fatigue and who fulfilled DSM-1V criteria for depression during the period of fatigue.

**Table 1:** Characteristics of individuals who fulfil different duration and associated symptom criteria of disabling fatigue

Characteristic	"3 months plus disabling fatigue"	"Child like CFS"	"Adult like CFS"
Number of affected individuals	11	15	34
Male: Female ratio	1:2.3	1:2.0	1:2.7
Age of onset in months (SEM)	117.5 (20.4)	130.5 (11.1)	146.6 (8.1)
No of Days per week affected (SEM)	6.2 (0.5)	4.6 (0.5)	5.8 (0.3)
Total No of hrs resting and sleeping per day (SEM)	13.7 (0.8)	13.3 (0.8)	13.1 (1.1)
Global impairment score (SEM)	5.9 (0.4)	6.5 (0.3)	6.5 (0.2)
No of days per term absent from school during worst period	18.8 (13.2)	17.3 (4.7)	12.9 (2.7)
% who contacted their GP with regard to the fatigue	55%	73%	71%
% with DSM-IV clinical depression	44%	52%	53%

SEM = standard error of mean

The results of the statistical analysis suggested no significant difference (P > 0.1) between the groups. The only exception to this was the number of days that the child was affected during the episode of fatigue, for which there is a significant difference between individuals who were classed as suffering from "adult like CFS" and those classed as suffering from "child like CFS" (t = -2.58, df 47, p = 0.027). These analyses were repeated using non-parametric tests, however there was no difference in results.

The descriptive statistics suggested that there was greater variability in individuals classed as suffering from "3 months plus disabling fatigue" than in individuals in the other two groups, particularly for the age of onset and number days per term absent from school. Less individuals from this group had visited a GP with regard to the fatigue. Although no significant differences or trends were found between individuals classed as suffering from "3 months plus disabling fatigue" and those classed as suffering from "adult like CFS" for these 3 variables, because of the descriptive statistics, a post hoc set of analyses was also conducted. For the post hoc analyses individuals classed as suffering from "adult like CFS" and "child like CFS" (i.e. all those individuals who had more than 3 months disabling fatigue and whose symptom profile resembled that of the CDC operational criteria for CFS) were combined into one group and compared to those classed as suffering from "3 months plus disabling fatigue". However no significant differences were found between these groups for age of onset, number of days absent from school per term or visits to the GP. As in the previous statistical analyses, these tests were also repeated using non-parametric tests however there was no difference in the pattern of results.

## Discussion

The results indicate that the clinical and demographic characteristics and level of impairment of children with narrowly defined disabling fatigue and more broadly defined fatigue are similar. There was no significant difference in gender ratio, the average age of onset or the rate of comorbidity with depression between the groups. Likewise affected young people seemed to spend a similar number of hours resting and/or sleeping, there was parental report of similar levels of global impairment, they were absent for a similar number of days from school during the term in which they were most affected by the fatigue and a similar percentage of individuals were contacting their GP about the fatigue.

A lack of significant findings cannot necessarily be interpreted as meaning there is no differences between these groups but there are still a number of implications of these results. Specifically, the results suggest that a longer duration of fatigue and accompanying symptoms are not necessary in defining disabling, impairing fatigue in children and adolescents. Children who present with shorter histories of fatigue and/or in the absence of CFS associated symptoms have high levels of parent reported impairment, both in terms of family and peer relationships and school work. They also appear to be absent for a substantial number of days from school and it causes enough worry for the parents to take the majority of these children and adolescents to a GP. Even though their was a significant difference for the number of days affected by the disabling fatigue between those individuals classed as suffering from "adult like CFS" and "child like CFS", all groups reported that they were affected by the disabling fatigue for most days of the week.

The descriptive statistics also suggested the possibility that individuals within the "three months plus disabling fatigue group" may be more heterogeneous, given that there is a relatively large amount of variance in this group in comparison to the other groups. Although this may have nosological implications it does not negate the high level of impairment this group also appears to be experiencing.

One important limitation of this study is that the individual's symptoms were identified by telephone interview rather than medical examination. Although every effort was made to exclude individuals who may have had an alternative diagnosis that would explain the fatigue this does not guarantee that if an in depth medical assessment were undertaken with these individuals that a number would not be classed as fulfilling the CDC definition of CFS. This may in some way explain why over half individuals in this population based sample showed a similar symptom profile to the CDC criteria for CFS whereas in the adult population only one third were categorised as fulfilling this criteria [17], although this may also be due to differences in presentation and/or aetiology of disabling fatigue between adults and children and adolescents. However this does not change the fact that all these individuals are reporting high levels of impairment and interference with their life due to the fatigue.

A further possible limitation is that parent report of disabling fatigue was used to identify individuals for further data collection and the analysis is based on information from parental interview only. There are often low levels of agreement between parent and child/adolescent report of behavioural/psychiatric symptoms [23] and there is some evidence that this is also the case with CFS symptoms [24]. However as life time ever disabling fatigue was being examined and as there is evidence that adolescents and young adults may often not recall key symptoms (for example, over 50% of individuals with a diagnosis of depression between 15–21 failed to recall a key symptom at age 25 [25]) it seemed most appropriate to use parent report. Further interviews with twins over the age of 12 were also conducted and on prompting 97% were able to recall a period of disabling fatigue about which it was possible to conduct the interview.

## Conclusion

These findings have clinical significance in that they imply episodes of fatigue in children and adolescents which last at least 3 months warrant investigation regardless of whether or not a diagnosis of CFS can be made. This is because the degree of impairment is substantial in terms of interference with usual activities, family and peer relationships, school absence and school work, and the impairment does not appear to differ from the level of impairment found in more narrowly defined disabling fatigue.

There was no indication that the group of children with shorter periods of fatigue differed on a number of associated characteristics (in terms of gender ratio, comorbidity with depression and age of onset) or in terms of impairment from the group of children who had a symptom profile similar to the CDC criteria for CFS. Although this does not prove that there is no differences between individuals in these groups it does indicate that within a primary care setting it may be appropriate to consider a broader definition of disabling fatigue in children for clinical and research purposes.

## Competing interests

The author(s) declare that they have no competing interests.

## Authors' contributions

TF interviewed the families, performed the statistical analysis and drafted the manuscript. PM provided clinical advice when conducting the analysis and helped drafted the manuscript. AT participated in the design and coordination of the study. AF conceived of the study, and participated in its design and coordination.

## Pre-publication history

The pre-publication history for this paper can be accessed here:


